# Adaptive Remodeling of the Neuromuscular Junction with Aging

**DOI:** 10.3390/cells11071150

**Published:** 2022-03-29

**Authors:** Michael R. Deschenes, Rachel Flannery, Alexis Hawbaker, Leah Patek, Mia Mifsud

**Affiliations:** Department of Kinesiology & Health Sciences, College of William & Mary, Williamsburg, VA 23187, USA; rachel.flannery@wm.edu (R.F.); alexis.hawbaker@wm.edu (A.H.); leah.patek@wm.edu (L.P.); mia.mifsud@wm.edu (M.M.)

**Keywords:** acetylcholine (ACh), nerve terminal, endplate, vesicle, sarcopenia, NMJ

## Abstract

Aging is associated with gradual degeneration, in mass and function, of the neuromuscular system. This process, referred to as “sarcopenia”, is considered a disease by itself, and it has been linked to a number of other serious maladies such as type II diabetes, osteoporosis, arthritis, cardiovascular disease, and even dementia. While the molecular causes of sarcopenia remain to be fully elucidated, recent findings have implicated the neuromuscular junction (NMJ) as being an important locus in the development and progression of that malady. This synapse, which connects motor neurons to the muscle fibers that they innervate, has been found to degenerate with age, contributing both to senescent-related declines in muscle mass and function. The NMJ also shows plasticity in response to a number of neuromuscular diseases such as amyotrophic lateral sclerosis (ALS) and Lambert-Eaton myasthenic syndrome (LEMS). Here, the structural and functional degradation of the NMJ associated with aging and disease is described, along with the measures that might be taken to effectively mitigate, if not fully prevent, that degeneration.

## 1. Introduction

Demographic data from throughout the world indicate that a growing segment of the population in western countries is considered aged with no signs of abatement of this trend in the future [1]. This trend is associated with increased healthcare costs in those nations as aging is known to incur a greater incidence of illnesses, injuries, and various ailments. Indeed, in the United States, although the aged (≥65 years) account for only 15% of the total population, they are responsible for 34% of total healthcare expenditures [2]. All physiological systems of the body are negatively affected by aging, but aging of the neuromuscular system is of particular concern. In part, this is explained by the fact that skeletal muscle makes up such a large portion of the body’s mass, i.e., 40% in men and 33% in women. Moreover, maintenance of proper function of the neuromuscular system is essential as voluntary contractile force is required not only for recreational activities such as dance and sports but also activities that are necessary for survival, i.e., eating, breathing, and evading danger. At the very core of the vertebrate neuromuscular system is the neuromuscular junction (NMJ), which is the vital synapse joining the excitatory messages of the motor nervous system with the contractile function of skeletal muscle [3,4,5]. Research has clearly demonstrated that aging causes gradual dysfunction of the vertebrate neuromuscular system, including the loss of contractile strength and power. This age-related decline in neuromuscular mass and function is referred to as “sarcopenia” [6], which first manifests at about 50 years of age, while its rate of degeneration noticeably accelerates beyond the age of 60 years [7,8]. In recent years, the NMJ has increasingly been linked to the onset and progression of sarcopenia [9,10,11]. Although it is unlikely that aging of the NMJ alone is the causative factor of sarcopenia, it is just as unlikely that it has no effect on age-related loss of neuromuscular function. The NMJ has not only been suggested to play a role in the onset of sarcopenia but also other age-related diseases such as amyotrophic lateral sclerosis (ALS), also known as Lou Gehrig disease. This is a fatal neurodegenerative disease that is usually detected in the eighth decade of life and is conventionally believed to originate in the motor neurons of the central nervous system (CNS) before proceeding to the skeletal muscle in an anterograde manner. However, more recently, it has been suggested that this disease may first manifest itself at the NMJ and propagate in a retrograde manner along the motor neurons’ axons in a “dying back” direction [12,13,14,15,16,17]. What is clearly known, however, is that the NMJ is affected by ALS, resulting in weakening and wasting of the skeletal muscle [12,13,14].

Lambert-Eaton myasthenic syndrome (LEMS) is another disease usually afflicting the aged, in this case by impairing the NMJ’s ability to properly function by preventing its release of the neurotransmitter acetylcholine (ACh). However, unlike ALS, this autoimmune disorder is not life-threatening, even though it causes significant muscle weakness [18,19]. The important role that the NMJ plays in maintaining proper neuromuscular function is illustrated by the above and other afflictions including myasthenia gravis, which causes deterioration of muscles of the face and limbs, along with the muscular dystrophies, which are a group of conditions causing muscle wasting and weakness [20,21,22].

**Figure 1:**
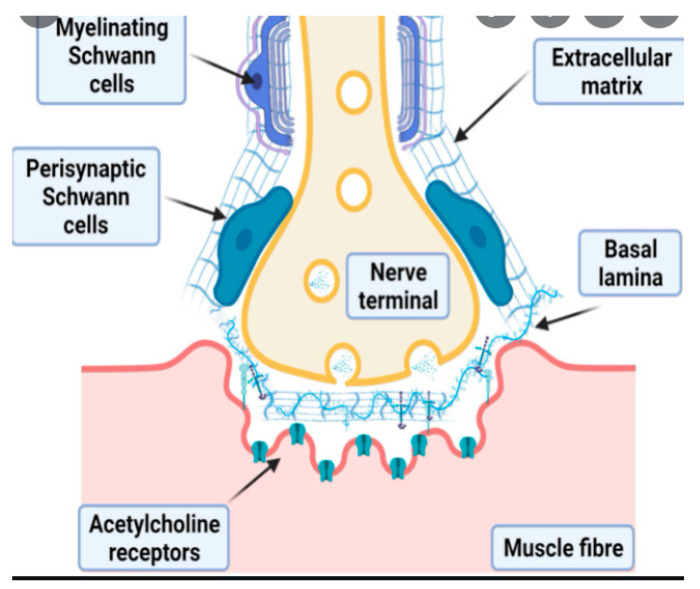
Positioning of the peri-synaptic Schwann cell at the neuromuscular junction.

Traditionally, the structure of the vertebrate NMJ has been described as a two-component model, i.e., the presynaptic nerve terminal of a motor neuron, and the postsynaptic endplate, which is a specialized region of the myofiber innervated by that motor neuron [23,24,25]. More recently, however, the NMJ has increasingly come to be thought of as a three-component synapse, wherein the additional constituent is the peri-synaptic Schwann cell, which is a glial cell that wraps around the nerve terminal ending and dips into the synaptic cleft [26,27,28] (Figure 1). This under-appreciated supportive cell plays a critical role in ensuring synaptic efficiency, by allowing electrical impulses carried by the presynaptic motor neuron to be effectively delivered to the postsynaptic myofiber, resulting in contractile activity. These specialized glial cells are also regulators of remodeling of the NMJ, both the subtle, steady version that occurs throughout life—where the NMJ displays observable plasticity—as well as the more pronounced remodeling that is associated with various stimuli such as exercise, disuse, natural growth and development and aging [29,30,31].

This tripartite synapse is unique in many ways, and because of its relatively simple arrangement and easy access, is often used as a model to study the synaptic function [23,26]. As examples of its specific features, the NMJ has a single presynaptic cell, i.e., the motor neuron, and a single postsynaptic cell, the myofiber. Moreover, only a single neurotransmitter is secreted, i.e., ACh, and under normal conditions, enough of that excitatory chemical agent is released into the synapse to elicit a postsynaptic response, or depolarization, to bring about a muscle twitch. In other synapses, the amount of neurotransmitter released in response to a single action potential is typically of sub-threshold intensity, failing to trigger a postsynaptic response.

Yet, at the NMJ, the amount of neurotransmitter released in response to a single presynaptic impulse is several-fold greater than is necessary to trigger a postsynaptic response. This excess amount of ACh released is referred to as the “safety factor” of neuromuscular transmission and does not occur at other synapses of the body. In viewing the NMJ’s role within the function of the larger neuromuscular system, it should be emphasized that although each myofiber responds to a single presynaptic motor nerve terminal, each motor neuron innervates a number of myofibers. Indeed, the functional component of the neuromuscular system is the “motor unit”, which comprises a single motor neuron and all the myofibers it innervates. Depending on the size of the motor neuron, and particularly the gauge of its axon, the number of myofibers comprising that motor unit may be as little as merely a few, as in the case of a thin axon, ranging to as many as hundreds for a thick axon. As can be easily imagined, the ultimate force produced by a contracting muscle largely depends on the number and size of the motor units activated. Since they are all innervated by the same motor neuron, the contractile and metabolic properties of those myofibers are shared by all of them, and they are categorized accordingly, i.e., all type I or II, all fast-glycolytic, fast-oxidative glycolytic, slow-oxidative, etc. Furthermore, recruitment of these various motor units during a voluntary contraction does not occur in a random fashion, but rather, can be easily predicted based on the “size principle” of motor unit recruitment. The “size principle” was first described by E. Henneman in 1957 [32], and was confirmed with greater detail in a series of classic studies conducted by R.E. Burke in 1967 [33]. Specifically, this principle states that smaller motor units—smaller in the number of myofibers, as well as the size of those myofibers—are recruited first, thus developing no more force than is necessary to complete the task at hand. Should more force be required, larger motor units are then progressively activated until the necessary contractile force is achieved. Since smaller motor units are characterized by greater aerobic metabolic capacity, i.e., increased mitochondrial content and capillarization, production of lactate and fatiguing metabolites is minimized until larger, glycolytic motor units are called into play. As one might expect, if myofibers are designed with different metabolic profiles that match them to the tasks they are assigned to complete, so too are NMJs designed to achieve their tasks.

## 2. Design of NMJs and Motor Units

The function of the NMJ begins with the motor neuron, the cell body (soma) of which resides in the central nervous system, where it initiates an electrical impulse, or action potential, which travels down the axon in an anterograde fashion to the skeletal myofibers that the neuron innervates. At the terminal end of the axon, myelination is no longer expressed around the axon, and instead, it branches out to a number of nerve terminal endings, which express voltage-gated calcium channels. On the arrival of the action potential at the nerve terminal ending, these voltage-gated channels are opened, resulting in a sharp, sudden influx of calcium into the terminal’s cytomatrix. This newly arrived calcium is sensed by the protein “synaptotagmin”, which is embedded in the membranes of presynaptic ACh containing vesicles [34,35]. By interacting with a series of SNARE proteins located both on the vesicular and terminal ending membranes, the binding of calcium with synaptotagmin releases vesicles from their “docked” positions at presynaptic “active zones”, which are positioned in direct apposition from postsynaptic ACh receptors distributed on the postsynaptic endplate [36,37,38]. The newly released vesicles then fuse with the terminal ending’s membrane, resulting in exocytosis of the ACh residing in the vesicles into the synaptic cleft separating the pre- and postsynaptic cells. From there, the neurotransmitter crosses the cleft via random diffusion and binds with ligand-gated, postsynaptic ACh receptors of the endplate region of the myofiber’s sarcolemma. Since ACh travels solely according to passive diffusion, the terminal of the neuron is only ~50 nm from the postsynaptic endplate, and the vesicles are released from sites in the nerve terminal that are in direct “apposition” or juxtaposed to postsynaptic receptors. This positioning maximizes the probability of diffusing ACh binding to those receptors and evoking a postsynaptic electrical response [24]. These two morphological details, i.e., limited width of the synaptic cleft, and apposition of ACh release sites from binding sites, are also vital characteristics of the NMJ, allowing it to serve as an efficient synapse. Importantly, also found in the synaptic cleft is the enzyme acetylcholinesterase (AChE), the functions of which are to destroy ACh bound to the postsynaptic receptors and remove debris resulting from that action. This permits control of the continuation or cessation of neuromuscular transmission.

**Figure 2:**
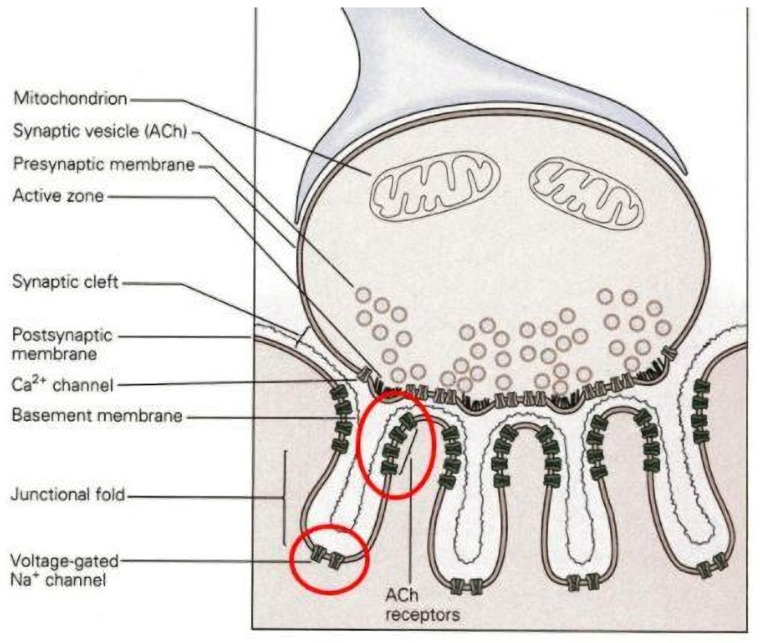
Illustration of the pre- and postsynaptic features of the NMJ; red circles indicate the presence of ligand-gated acetylcholine receptors at the crests of postsynaptic junctional folds, and voltage-gated sodium channels in the depths of those folds.

At the postsynaptic side of the NMJ is the endplate, which is a specialized, swollen region of the myofiber’s sarcolemma that accounts for only one-tenth of one percent (0.1%) of the entire surface of the sarcolemma. Etched into the thickened region of the endplate are the “junctional folds” of the membrane. On the crests of these infoldings are a great number of ACh receptors—the folded nature of the membrane permits many receptors to be expressed at the endplate, contributing to the transmission safety factor we earlier alluded to—while in the depth of these junctional folds are voltage-gated sodium channels (Figure 2).

Details of the process of neuromuscular transmission show that on the binding of ACh receptors by its neurotransmitter, a conformational shift occurs at the receptor, allowing ion flux into the myofiber’s cytosol—the receptor is both a binding site and an ion channel—inducing “endplate potential” (EPP). This depolarization is a graded response as the channels are ligand- and not voltage-gated, so that the strength of the depolarization is proportional to the amount of ACh bound to its receptors, rather than being an “all or none” event, as observed in voltage-gated channels. This EPP is confined to the area of the endplate region, but it diffuses into the depths of the junctional fold where voltage-gated sodium channels are located. Due to the “safety factor” of the EPP, as described earlier, a single EPP will normally be of more than sufficient intensity to open the voltage-gated sodium channels located on the sarcolemma—including the depths of junctional folds—thus evoking an action potential. This all-or-none impulse then spreads throughout the rest of the sarcolemma, delving into the T-tubules, where it will excite the “dihydropyridine (DHP) receptors”, which act as voltage sensors that will then stimulate “ryanodine receptors” located on the membrane of the myofiber’s sarcoplasmic reticulum. In response, the channel that is part of the ryanodine receptor opens, allowing an efflux of that internally stored calcium, to increase the levels of cytosolic calcium within the myofiber. This newly-released calcium will bind to troponin, leading to actomyosin cross-bridge formation and muscle contraction. This contractile activity continues as long as there is effective neuromuscular transmission at the NMJ. Typically, it ends, as mentioned earlier, when AChE cleaves neurotransmitters bound to endplate ACh receptors without replacing these through continued release of ACh from the motor neuron. This entire process has been well described elsewhere [39,40].

## 3. Effects of Aging on the NMJ

Earlier in this report, the negative consequences of certain diseases on the NMJ were described. These afflictions are generally observed in older individuals. This, of course, begs the question of whether aging itself alters the NMJ’s structure and function. Although it is often difficult to separate the effects of aging from age-related diseases, there is ample evidence that even in the absence of disease, aging conveys deleterious effects on the NMJ. This validates the idea that the NMJ undergoes constant remodeling throughout the lifespan, and that such activity is significantly increased in those of advanced age [41,42,43]. We will now turn our focus to the morphological adaptations of the NMJ to aging.

### 3.1. Morphological Adaptations

There is a considerable body of evidence that senescence is associated with significant remodeling of the NMJ (recently reviewed by Pratt et al., 2021; Dobrowolny et al., 2021; Iyer et al., 2021). For example, aging has been found to bring about enhanced presynaptic nerve terminal branching, both as revealed by an increase in the total length of nerve terminal branches [43,44,45], and the number of terminal branches present at the NMJ [44,45,46]. Moreover, the complexity of this branching—with essentially, the total branch length as a factor of the branch number (please see figure legend for details)—has been found to be greater among aged nerve terminals than young ones [47,48,49,50]. It has been suggested that this indicates greater remodeling of the motor neuron in an attempt to improve communication between pre- and postsynaptic components of the NMJ, as such communication is pared down with aging [9,51].

**Figure 3:**
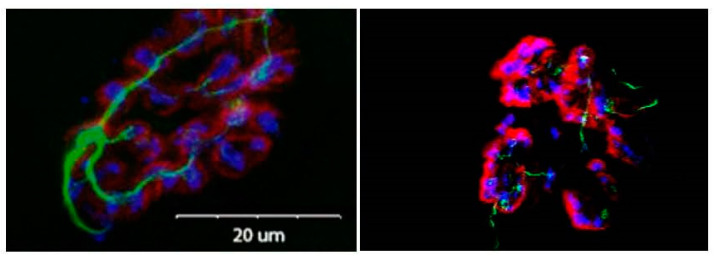
Micrograph showing close coupling of presynaptic vesicles and terminal branches with postsynaptic receptors. Presynaptic terminal branches are stained green, presynaptic vesicles are stained blue and postsynaptic receptors are stained red. Note the greater complexity of nerve terminal branching in aged NMJ.

In addition to the expansion of the nerve terminal network, aging has also been shown to decrease the total number of presynaptic, neurotransmitter-containing vesicles held by those terminal branches [52,53,54]. This is accompanied by reduced expression of the active zones where Ach-containing vesicles are docked, awaiting their release with the influx of calcium into the terminal [55]. This change in active zone number is precipitated by the selective degeneration of proteins that comprise the active zone, such as Bassoon, Piccolo, and voltage-gated calcium channels [55,56]. It is likely that the reduced presence of these active zone proteins is a consequence of the impaired axonal transport noted among aged NMJs [57,58]. This impaired axonal transport would hinder the replacement of those recently deteriorated proteins, which were initially generated in the neuron’s soma and subsequently transported to the locations where they were needed at the terminal endings. Similarly, it is reasonable to suggest that the reduction in vesicular-stored ACh at aged NMJs also comes about due to this interrupted axonal transport, and that this would account for the diminution of pre- to postsynaptic communication detected among senescent NMJs. Despite the significant increase in the total length and complexity of aged nerve-terminal branching patterns, the fidelity of the juxtaposition, or coupling (Figure 3) of pre- to postsynaptic binding sites, is maintained throughout the lifespan, seemingly as an attempt to maintain communication despite gross morphological alterations [59]. This ability to maintain pre- to postsynaptic coupling may be attributed, at least in part, to calcium channels that physically tether the postsynaptic endplate to the presynaptic active zones of the nerve terminal [60]. That said, there are data indicating that aging is associated with a lesser degree of pre- to postsynaptic coupling [46,61]. These inconsistencies in results may well be explained by how coupling was assessed, i.e., vesicle with receptors, branches with endplate area, etc. Moreover, despite alterations to the length and complexity of nerve-terminal branching, the number of ACh-containing vesicles per given length of branch is unaffected by aging [62,63].

**Figure 4:**
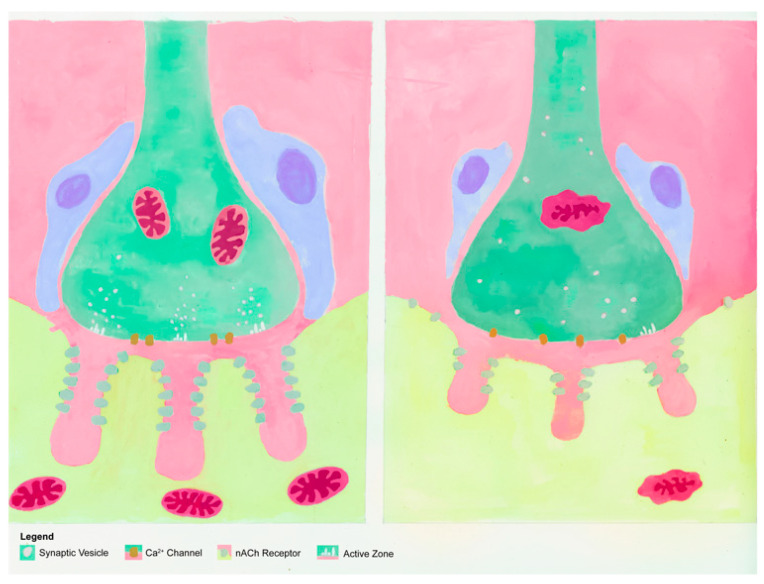
Illustration of aged and young adult endplates. Note more shallow gutters in the aged neuromuscular junction, with fewer receptors.

The effects of aging are also evident in the postsynaptic component of the NMJ. In general, it is fair to say that aging is associated with an expansion of postsynaptic features. More specifically, aged NMJs display larger total endplate areas, along with greater dispersion of ACh receptors within those endplates [46,61,64,65,66]. This dispersion is also referred to as “fragmentation” of the endplate [67,68]. At the same time as this fragmentation occurs, there is a decrease in the number of ACh receptors in aged NMJs, which may be the result of more shallow junctional folds at the endplate (Figure 4), thus reducing the surface area that postsynaptic receptors may be distributed within [69,70,71]. Aging is also associated with a reduction in the content of healthy mitochondria below the surface of the endplate [54,72,73]. These fewer and damaged mitochondria are known to release reactive oxygen species (ROS), which have a destructive effect on proteins and nucleic acids [72,73,74,75].

**Figure 5:**
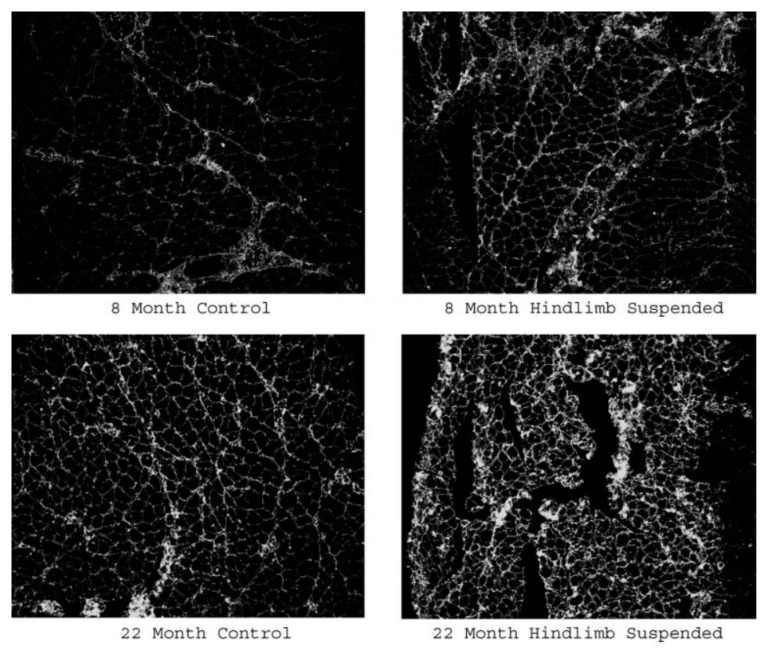
NCAM expression of aged (22 mo) and young adult (8 mo) muscle fibers under either control or unweighted (hindlimb suspended) conditions. Note that the NCAM expression is greatest in aged, unweighted fibers. Source: Deschenes and Wilson (2003).

As a further sign of the gradual denervation that occurs with aging, thus leading to NMJ remodeling, it has been reported that aged myofibers express greater amounts of the neural cell adhesion molecule (NCAM) than younger fibers (Figure 5). This supports the notion that remodeling of the NMJ is a lifelong process that is enhanced, but not initiated, by aging. The NCAM is a synaptogenic molecule expressed by denervated tissue as it has chemotactic effects that draw nerve-terminal endings to sites where the NCAM is expressed [76,77,78].

It is important to note that although aging is generally believed to have an expansive effect on the NMJ, there are data showing that in highly aged muscle tissue, there are decreases in the dimensions of the NMJ, both at pre- and postsynaptic sites, bringing about what is considered a dichotomous response to increasing age, i.e., expansion with early stages of senescence, followed by an atrophying effect with advanced senescence [49,64,79]. These conflicting findings may be related to the fact that many studying the effects of aging simply assume that all “aged” subjects are the same. Yet, it is far more likely that a modestly aged subject will demonstrate different characteristics and responses to someone severely aged. Indeed, in human studies, gerontologists have clarified three different subcategories of the aged, from “youngest-old”: 65–74 years of age, to “middle-old”: 75–84 years of age and “oldest-old”: ≥85 years of age [80]. This difference of what can be expected at different stages of senescence should be borne in mind by all investigators studying the effects of aging. To date, it has been consistently reported that with aging comes a decreased capacity for terminal sprouting in peri-synaptic Schwann cells, as well as heightened sensitivity to inflammation of those same cells, leading to the diminished regenerative capacity of aging neurons [16,81]. Moreover, 80% of aged endplates are no longer in contact with peri-synaptic Schwann cells, while only about 20% of aged NMJs are fully capped by those glial cells as they are in young adult synapses [16,82]. Those peri-synaptic Schwann cells that are seen at aged NMJs typically exhibit unusually thin, disorganized terminal branches/sprouts.

Interestingly, much like the muscle fibers on which they reside, the effects of aging on NMJs may be fiber-type specific. For example, Rosenheimer and Smith (1985) found that aging was linked to a decrease in the total and average lengths of presynaptic nerve-terminal branches, as well as less branch sprouting potential in the primarily fast-twitch EDL muscle, while no such remodeling was detected in aged, mainly slow-twitch soleus muscles. Another study featuring a different perspective examined the effect of the fiber type within a single mixed-fiber-type muscle, i.e., the diaphragm muscle. The results indicated that type IIX and IIB myofibers of the diaphragm displayed greater numbers of nerve terminal branches with greater lengths. In contrast, the NMJs of type I and IIA fibers failed to display any apparent age-related changes in nerve-terminal branching or sprouting [46].

In another examination into the effects of the myofiber type on aging among NMJs, Deschenes et al. (2011) reported the effects of aging on NMJs from different fiber types on locomotor muscles. They showed that in the mainly slow-twitch soleus muscle, aging was linked with a greater nerve-terminal branch length, whereas fast-twitch fibers in that same muscle were associated with shorter branches and a reduced postsynaptic endplate area. Overall, these results suggest that aging-induced remodeling of the NMJ is fiber type-specific. Typical age-related morphological adaptations of the NMJ are presented in Table 1.

### 3.2. Physiological Adaptations

#### 3.2.1. Presynaptic

In addition to anatomical remodeling, aging triggers adaptations to the physiological function of the NMJ. For example, aged NMJs have been found to demonstrate an increase in the amount of ACh released from nerve terminals on their stimulation [50,83,84]. This suggests that more vesicles are released with the arrival of an action potential at presynaptic nerve-terminal endings, or that the quantal size—the amount of ACh stored per vesicle—is amplified with aging. Since the EPP, an indirect measure of quantal size, has been reported to be greater among aged NMJs, at least some of the gain in quantal content, or the amount of neurotransmitter released on stimulation, must be attributed to an increased quantal size or greater ACh per vesicle [14,50,85]. This increased quantal size is evident in data revealing that the total amount of ACh at nerve terminal endings is less among aged, than it is among young, adult NMJs [86,87,88]. Instead of having greater amounts of ACh to release, the likelihood of releasing a neurotransmitter on stimulation is more pronounced among aged NMJs [87,89]. This increased probability of vesicle release on stimulation is linked to the fact that with aging comes increased dysfunction of nerve-terminal calcium channels, allowing both a greater than normal influx of calcium on stimulation, as well as magnified non-stimulated leakage of calcium into the region of the vesicle anchoring active zones [90,91,92]. In effect, aging is associated with greater calcium influx into nerve terminal endings, both with stimulating and non-stimulating conditions, and by extension, a greater amount of ACh release, both during stimulation and at rest. In brief, aging results in a greater quantal content (amount of ACh released with a single impulse) and quantal size (amount of ACh stored in a single vesicle). This amplified release of ACh on stimulation, however, is also responsible for a greater rate of “run down” or decline in the amplitude of postsynaptic potential during a train of stimuli, leading to a faster rate of failure to stimulate adequate postsynaptic potential to elicit muscle contractile activity [93]. In short, then, the more pronounced release of ACh on stimulation early during a consecutive series of stimuli leads to a faster rate of neuromuscular fatigue, along with failure to execute the desired task. It is also of interest that recent findings indicate that not only is aging a factor in the onset of neuromuscular fatigue in continuously stimulated NMJs but it also interferes with post-activity recovery of neuromuscular function [94].

Another meaningful consequence of aging at the NMJ is a reduction in the safety factor during neuromuscular stimulation. Recall that the “safety factor” refers to the several-fold greater than needed amount of ACh released on stimulation, to elicit a postsynaptic muscle twitch. It is the increased nerve-terminal branching combined with the decreased number of presynaptic ACh vesicles that accounts for this—decreased safety factor—deleterious adaptation, which, in turn, contributes to the earlier onset of neuromuscular fatigue seen among the aged [89,95]. This evidence gives credence to the biological tenet that changes in form and function are inextricably linked.

#### 3.2.2. Postsynaptic

Despite the popularity among biologists of this adage of form and function being tightly linked, there are new findings suggesting that perhaps this bond between form and function is not as powerful as previously suggested. Recently, it has been reported that the fragmentation of the postsynaptic endplate—considered to be a hallmark of the aging NMJ—is not necessarily indicative of disrupted neuromuscular transmission. In data newly reported by Dr. Clarke Slater’s laboratory, it was found that the endplates of aged diaphragm muscles did, in fact, demonstrate increased fragmentation relative to those of young adult mice [48,67]. Yet, despite this exaggerated fragmentation, when these diaphragm muscles were assessed for neuromuscular transmission, no age-related differences were detected. That notwithstanding, an even more recent review of this topic asserts that disruption in the structure of the NMJ in aged muscles is indeed coupled with significant disturbances in their function [14,48,85].

**Figure 6:**
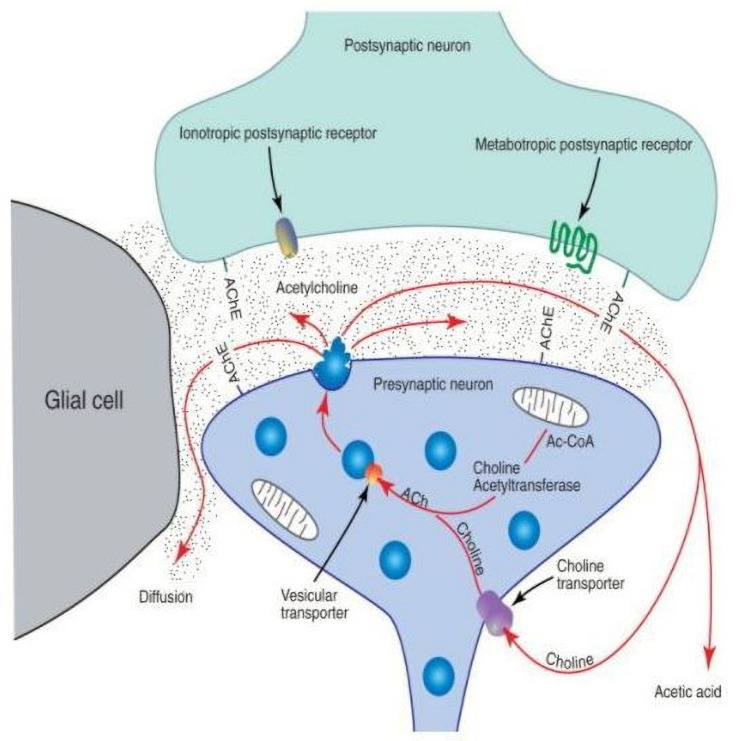
Synthesis of ACh by choline acetyltransferase at the presynaptic terminal.

Increasingly, mitochondrial abnormalities are viewed as having a causative effect for impairments of NMJ function. Both the presynaptic nerve-terminal ending and the postsynaptic endplate region are richly endowed with mitochondria [72,96,97], providing vast amounts of the ATP that is required for effective neuromuscular transmission. At the presynaptic nerve terminal, ACh is produced by choline acetyltransferase (ChAT) joining the acetyl group from acetyl co A, released by mitochondria, to choline released by the actions of AChE at the synapse (Figure 6). Thus, if mitochondrial synthesis of ATP is hindered, so will be the production of ACh, and by extension, the efficacy of neuromuscular transmission at the NMJ. These facts become particularly meaningful in the aging NMJ where ChAT is less plentiful and the membranes of resident mitochondria become fragile and subject to rupture. Such actions have the double effect of hindering ACh synthesis and enhancing the production of protein-damaging free radicals, or ROS. This relationship is yet another example of how changes in the form alter the function in biological settings.

Another indication of the importance of mitochondria in maintaining the NMJ’s structure and function is the impact of PGC-1α on the myoneural synapse. PGC-1α acts as a co-factor in mitochondrial transcriptional activities and thus helps assure normal mitochondrial metabolic activities and protection against ROS release. A recent investigation has found that adding PGC-1α to aged muscle reduces the ROS levels and restores a normal NMJ structure and function, obviating the effects of aging [98,99]. In addition to damage to mitochondrial membranes, other mechanistic factors contribute to age-related degeneration of the NMJ. For example, aging is accompanied by increased systemic inflammation, sometimes referred to as “inflammaging” [100]. Accordingly, there are elevated concentrations of inflammatory agents such as tumor necrosis factor-1 (TNF-1) and interleukin-6 (IL-6), which in part elicit degenerative actions on NMJs and the muscle tissue they innervate [100,101]. In a contrary manner, insulin-like growth factor-1 (IGF-1) is an anabolic agent primarily produced and released by skeletal muscle, which progressively declines with aging. It appears that the natural decline of this peptide contributes to the effects of aging on the NMJ, as restoring normal, youthful levels of IGF-1 re-establishes the structure and function of aging NMJs [35].

Another consideration for explaining age-related disruption to the function and form of the NMJ is altered expression of the postsynaptic protein laminin. This protein, in effect, serves as the foundation on which the postsynaptic endplate is assembled. In particular, it has been posited that laminin-α4 is vital to the alignment of postsynaptic receptors in direct apposition to presynaptic vesicles, thus maintaining proper pre- to postsynaptic coupling, and by extension, neurotransmission [102,103]. Much as with other proteins of the NMJ, aging results in a significant decline in laminin-α4, so it is not surprising that affected NMJs suffer structural and functional disruptions, including decrements in muscle strength [104,105]. Recent work has confirmed the importance of the proper expression of synaptic laminin [106]. In that study, aged mice with damaged NMJs were administered exogenous laminin-α4 to restore normal, youthful levels, and in doing so, similarly restore the NMJ structure, function, and even muscular strength [104]. Clearly, methods enabling proper pre- to postsynaptic coupling must be addressed in clinical procedures aimed at maintaining or improving the neuromuscular function among the aged.

Perhaps the most surprising player in the age-related degeneration of the NMJ is the very neurotransmitter that is used to power neuromuscular transmission. Although it is well understood that ACh functions as an excitatory neurotransmitter that enables neuromuscular transmission and muscular contraction, less appreciated is the fact that the same ACh exerts a degrading effect on postsynaptic endplates, thus disrupting normal neuromuscular transmission [71,107,108]. Accounting for the fact that typical nerve-to-muscle transmission, featuring the release of ACh onto the postsynaptic endplate, does not cause disruption of the working NMJ, is evidence that during such neuromuscular activity, the protein agrin is also released by nerve terminals onto the endplate, neutralizing the destructive effects of ACh [71,109]. In effect, the positive effects of agrin balance the negative effects of ACh at the NMJ.

Yet, with aging, this balancing capacity becomes impaired. Recall that aging is associated with an increased quantal content, or ACh release, on stimulation, which then yields a greater degenerative effect relative to the synaptic stabilizing effect of agrin [109,110]. In addition to a greater amount of ACh being released per impulse in aged NMJs, it has been determined that less agrin is released by those same NMJs. In concert, these two age-related adaptations provoke a gradual degradation of the NMJ. Confirming the roles played by ACh and agrin in giving rise to the aged NMJ is evidence that mice genetically engineered to secrete less ACh on stimulation also display fewer, less pronounced signs of aging, both in morphology and neuromuscular transmission [71]. Although these findings are both informative and exciting, much more work is needed to fully reveal the mechanisms involved in NMJ disturbances noted among aged muscle tissue. Common physiological alterations of the NMJ to aging are found in Table 2.

### 3.3. Countering Aging-Related NMJ Adaptations

It has been recognized for a number of years that a slow, steady process of remodeling of the NMJ occurs throughout life [111,112]. This typically features nerve-terminal branch withdrawal from specific sites within the postsynaptic endplate, only to re-probe and re-innervate that same myofiber at a different location of the same endplate [42,49,113]. In general, such subtle and slow remodeling of the NMJ imparts no functional alterations at that synapse [114]. With aging, however, this process of NMJ remodeling occurs at a more rapid rate and with a more pronounced severity. resulting in first partial, and then total, denervation of the affected myofiber, ultimately leading to its death, i.e., “sarcopenia” [115,116]. Some but not all of these newly abandoned, or denervated, myofibers will be re-innervated by neighboring motor neurons, although the requisite terminal sprouting is limited with aging [117,118].

A host of serious maladies are associated with sarcopenia—which itself is considered a disease—including diabetes, arthritis, and even some forms of cancer [101,119]. Clearly, the ability to control myofiber denervation, and thus sarcopenia, has serious health implications. To date, however, only two interventions have been reported to effectively manage age-related NMJ destruction: exercise and calorie restriction [120,121]. While dietary restriction has been shown to be effective in offsetting age-related NMJ deterioration, it requires a severe (40%) reduction in the daily calorie intake [122]. Such dietary restriction, while effective, is not a practical tool to combat NMJ destruction and sarcopenia in humans. However, there is a strong body of evidence that regular exercise training of even a moderate intensity effectively slows, if not fully prevents, age-related NMJ damage [123,124].

More recent, in-depth research delving into the mechanisms by which exercise and a restricted calorie intake attenuate the negative effects of aging on the NMJ has been informative [121,122]. For example, it has been determined that a limited dietary intake in rats results in a lower percentage of endplates found to be fragmented, with a smaller fraction of all endplates fully denervated, a smaller number of NMJs exhibiting nerve terminal sprouting, and even a decreased incidence of axonal atrophy. By comparison, exercise training was found to provide the same type of synapse protective effect as calorie restriction, but to a less pronounced degree. It was also revealed that with exercise training, only those muscles actively recruited during training sessions displayed anti-aging effects at the NMJ. This is unlike calorie restriction, where all muscles of the body experienced beneficial effects at the NMJ. It should also be noted that the beneficial effects to the NMJ of a reduced calorie intake acted principally by slowing the progress of natural neuronal decay and not by preventing or reversing the impact of aging. More specifically, calorie restriction imparts its positive effects on the NMJ by mitigating age-related death, or apoptosis, of motor neurons, i.e., the synaptic rescuing effects of calorie restriction are secondary to the sparing of aged motor neurons. Conversely, the advantageous outcomes of exercise on aging NMJs can actually reverse senescence-related damage to the NMJ function and structure. So, while both are effective, data suggest that a restricted calorie intake and exercise training elicit their NMJ sparing effects using different physiological mechanisms.

Recall that remodeling of the NMJ during aging displays two distinct phases. That is, in earlier stages of senescence, aging is characterized by expansion of the pre- and postsynaptic elements of the NMJ, with increased and more elaborate nerve-terminal branching patterns, along with an increased area of the postsynaptic endplate, accompanied by greater dispersion of Ach-containing and -binding, respectively, vesicles and receptors [41,56,121].

However, in later stages of aging, i.e., ≥25 months in murine muscle, a reduction, and not an expansion, of the NMJ is evident [49,64,79]. This is observed in both pre- and postsynaptic features of the NMJ. Although exercise is also effective in countering the effects of extreme aging at the NMJ, as there is a different baseline morphology than in moderately aged NMJs, adaptations to exercise training are different. Specifically, people who are very old display an NMJ size that is similar to, if not smaller than, that found in young, adult muscle. Accordingly, in these more modestly sized synapses, exercise training works to enhance the dimensions of presynaptic nerve-terminal branching and vesicle content, while simultaneously increasing the size of the postsynaptic endplates and the number of ACh receptors bound to those endplates [125,126]. Despite overall restructuring of the NMJ with exercise training among the aged, pre- to postsynaptic coupling remains intact [45,53].

In sum, in younger muscle, exercise training—whether it is endurance training, or resistance training, i.e., weightlifting—increases pre- and postsynaptic dimensions of the NMJ, without impacting the density of vesicle or receptor expression. Yet, evidence suggests that endurance training elicits a greater hypertrophic response, i.e., ~30% vs. ~15%, which is presumably related to the long-term continuous neuromuscular activity that characterizes endurance but not resistance training.

As much as an increase in neuromuscular activity can affect aged NMJs, so can a reduction in such activity. This reduced activity can present as immobilization, such as when wearing a cast following a bone fracture, or muscle unloading, such as having to participate in ambulatory activities while using crutches, or even being confined to bed rest. These are all considered forms of subtotal disuse, while total disuse is imparted by the use of neurotoxins, or manually crushing or severing a nerve leading into a muscle.

Any of these forms of disuse elicits a decline in muscle strength that is commonly accompanied by a decreased neural drive to contract, as assessed by electromyography (EMG) [127,128]. In studies featuring human participants and with superimposed electrical-stimulation procedures, it appears that the decreased neural drive stems from disuse-induced reductions in the neural drive of the central nervous system, and not from a neuromuscular block at the NMJ [129,130]. When examining the morphology of the NMJs in rodents subjected to muscle unloading, it was demonstrated that disuse interventions of two weeks do not alter the NMJ’s morphology or function in young adult or aged NMJs, although significant myofiber atrophy is apparent [63,131].

These findings clearly suggest that NMJs are more resistant to the deleterious effects of disuse than myofibers are, and that this is true in young adult and aged neuromuscular systems. However, an effect of aging on the consequences of disuse manifests when the duration of disuse is increased. More specifically, when young adult and aged rats underwent four weeks of unloading, aged but not young adult NMJs displayed typical signs of degeneration [132]. This was in addition to the signs of synaptic damage already evident in rats of the same age but living in control conditions. In effect, disuse worsened the negative adaptations already apparent in aged NMJs. In a similar vein, the disuse also amplified the myofiber atrophy noted among aged rats as a result of sarcopenia. Clearly, disuse imposed on aged neuromuscular systems provokes more severe maladaptation than in young adult ones, which suggests that we must do whatever is possible to minimize the chances of neuromuscular disuse among those that are senescent. This is particularly true given the slow rates of post-disuse neuromuscular recovery observed among the aged [94,132].

## 4. Conclusions

The evidence clearly suggests that aging negatively affects both the structure and function of the NMJ. This age-related remodeling of the NMJ’s morphology and physiology is rooted in the lifelong pattern of denervation and re-innervation of the motor endplate of the myoneural synapse. In younger synapses, these two processes—denervation and re-innervation—balance each other so that no significant disturbance in the NMJ’s form or function is evident. However, with aging, this balance becomes skewed such that denervation events exceed those of re-innervation, leading to the NMJ’s degeneration. Indeed, the importance of maintaining a proper balance between denervation and re-innervation at the NMJ was recently emphasized [133] by data demonstrating that those myofibers expressing synapses no longer capable of striking a proper balance are destined to die via denervation-induced sarcopenia, thus increasing the risk of incurring one of many health conditions such as diabetes or cancer. If the impact of aging on the NMJ is not by itself serious enough, aging also exacerbates the detrimental effects of disuse both on the NMJ and the myofiber it innervates, and may even dampen the positive effects typically induced by exercise on the neuromuscular system. Obviously, significant health benefits would be gained by effectively ameliorating the negative effects of aging on the NMJ. To date, however, only exercise training and calorie restriction have proven successful in those efforts.

## Figures and Tables

**Table 1:** Morphological Adaptations of the NMJ to Aging.

**Presynaptic**	**Authors**
Increased complexity of nerve terminal branching	Khosa et al., 2019; Andonian and Fahim, 1989; Fahim, 1997
Increased nerve terminal branch number	Deschenes et al., 2020; Prakash and Sieck, 1998; Deschene et al., 2010
Increased total nerve terminal branch length	Robbins and Fahim, 1985; Deschenes et al., 2020; Deschenes et al., 2016
Increased planar area of nerve terminal branch length	Fahim, 1997; Prakash and Sieck, 1998
Increased area of vesicle clusters	Deschenes et al., 2011; Deschenes et al., 2010
Decreased total number of vesicles	Deschenes et al., 2015; Taetzch and Valdez, 2018
Decreased number and concentration of active zones	Nishimune et al., 2016
**Postsynaptic**	**Authors**
Abandoned incidence of abandoned endplate gutters	Rosenheimer and Smith, 1985; Bao et al., 2020
Increased fragmentation of receptors	Willadt et al., 2016; Deschenes et al., 2015; Hunter, 2016
Decreased length of endplate	Vaughn et al., 2019; Arnold et al., 2014
Decreased total area of endplate	Prakash and Sieck, 1998; Fahim and Robbins, 1982
Decreased perimeter length around endplate	Jang and Van Remmen, 2011; Elkerdany and Fahim, 1993
Increased expression of NCAM	Deschenes and Wilson, 2003

**Table 2:** Physiological Adaptations of the NMJ to Aging.

**Presynaptic Alterations**	**Authors**
Increased quantal content	Fahim, 1997; Alshuaib and Fahim, 1990; Mahoney et al., 2014
Increased quantal size	Fahim, 1997; Jones et al., 2016
Increased spontaneous release of ACh	Smith, 1984; Smith and Weiler, 1987
Decreased calcium clearance from nerve terminal	Smith, 1987
**Postsynaptic**	**Authors**
Increased endplate potential amplitude	Iyer, 2021; Smith, 1987
Reduced safety factor of endplate potential	Giovannini et al., 2002; Liu et al., 2019
Increased synaptic depression during train of stimuli	Feng and Dai, 1990
Increased incidence of neurotransmission failure	Fahim, 1997; Smith and Weiler, 1987

## Data Availability

Not applicable.
